# Murine intestinal epithelial cells and lymphocytes undergo contrasting inflammatory shifts during gastrointestinal development

**DOI:** 10.1038/s41390-025-04262-z

**Published:** 2025-07-21

**Authors:** Thomas C. Wiemers, Jan Riedel, Niklas Dressler, Xiaoyan Feng, Nicole Peukert, Martin Lacher, Steffi Mayer

**Affiliations:** Department for Pediatric Surgery, University Hospital of Leipzig, Leipzig, Saxony Germany

## Abstract

**Background:**

The gastrointestinal tract is particularly vulnerable to strong inflammatory responses during early development as seen in preterm infants with necrotizing enterocolitis (NEC). Intestinal maturation plays a crucial role in the pathogenesis of this condition.

**Methods:**

Given the limited availability of human samples across different stages of gastrointestinal maturation, this study utilised a murine model that closely mirrors the fetal, preterm, term, and adult stages of human development. We investigated baseline and lipopolysaccharide (LPS)-induced inflammatory responses in isolated primary intestinal epithelial cells (IECs) and intraepithelial lymphocytes (IELs).

**Results:**

IECs displayed greater sensitivity to LPS at early developmental stages, with reduced responsiveness as maturation progressed. In contrast, IELs exhibited inflammatory reactivity only at later stages. Regulation of phosphorylated p65 in both cell populations highlighted the role of the TLR-4/NFĸB pathway in these maturation-dependent responses.

**Conclusion:**

A proinflammatory shift in key epithelial cell populations was observed, reflecting the development of the gastrointestinal system. These findings enhance the understanding of NEC pathogenesis and provide translational insights into intestinal inflammatory responses during maturation.

**Impact:**

- Intestinal epithelial cells (IECs) and intraepithelial lymphocytes (IELs) exhibit contrasting inflammatory responses during gastrointestinal maturation, with IECs being more reactive in early stages and IELs becoming reactive later.
- This study provides a detailed correlation between human and murine intestinal development, offering insights into maturation-dependent inflammatory mechanisms and the role of the TLR-4/NFĸB pathway.
- Our findings enhance understanding of gastrointestinal maturation and its role in inflammatory diseases like necrotizing enterocolitis (NEC).

## Introduction

Necrotizing enterocolitis (NEC) is a severe inflammatory condition predominantly affecting preterm infants that is characterized by intestinal tissue damage and high mortality rates.^1^ Establishing stable microbiome homeostasis is vital to prevent excessive inflammatory responses to colonizing microflora.^2^ Despite ongoing research, the exact mechanisms of NEC remain unclear. Progress is hindered by the scarcity of human samples, the variability of experimental animal models, and their limited comparability to the human condition.

Immaturity of the intestinal epithelial layer is linked to a high expression of the Toll-like-Receptor 4 (TLR-4) and increases NEC risk in preterms.^3^ TLR-4 belongs to a family of pathogen-associated-molecular-pattern (PAMP) receptors that recognizes along with its co-receptor myeloid differentiation factor 2 (MD-2), microbial ligands such as lipopolysaccharide (LPS) and activates both the innate and adaptive immune responses, thus promoting inflammation.^4^ LPS is a central cell wall component of gram-negative bacteria that induces inflammation, reduces transepithelial resistance, and increases the rate of apoptosis.^5,6^ Stimulation of TLR-4 by LPS leads to the activation of NFκB and its subunit p65, which translocates into the nucleus and promotes the transcription of proinflammatory cytokines and chemokines.^7,8^ Mice with deficient TLR-4 expression in their intestinal epithelial cells (IECs) exhibit resistance to NEC, and in both experimental and human NEC, significantly elevated TLR-4 expression is noted. Notably, inflammatory conditions cannot be induced in older mice, underscoring the importance of gut immaturity in NEC pathogenesis in mice and humans.^9,10^

The limited availability of human intestinal tissue for studying developmental processes necessitates alternative experimental systems with high similarity to human tissue. A fundamental requirement for these systems is a high degree of similarity to the human reference source. While routinely used laboratory cell lines can provide valuable insights, they typically reflect a specific maturation stage and exhibit stage-specific cellular behaviour. This poses challenges when comparing two or more different maturation stages, particularly if the available cell lines do not share the same origin. In the multitude of experimental studies on NEC, only few have attempted to establish direct correlations between developmental stages in mice and humans yet.

**Fig. 1:**
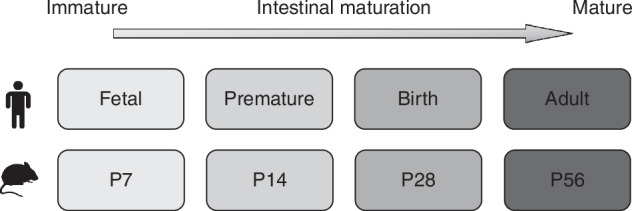
Correlation of murine and human stages of intestinal development. Based on homeostatic, structural, epithelial-specific gene expression pattern as well as cell appearance gastrointestinal stages are comparable between mice at P7 (one week), P14 (two weeks), P28 (four weeks), and P56 (eight weeks) and fetal, preterm, term, and adult human intestines.^11^

This study seeks to achieve a more precise temporal resolution of the LPS-induced inflammatory response at the critical interface between the microbiome mimicked by LPS stimulation, and the intestine by analysing primary IECs and intraepithelial lymphocytes (IELs) from different age groups. The latter were chosen according to Stanford et al. that compared gene expression and cellular characteristics under homeostatic conditions to establish age equivalency in mouse models to human.^11^ Accordingly, murine intestines at P7 correspond to fetal, at P14 to preterm (22–23 weeks), at P28 to term, and at P56 to adult human tissue (Fig. 1).

Based on Stanford’s suggestions, we systematically studied the inflammatory response of murine primary IECs and IELs, with and without LPS stimulation, during various stages of gastrointestinal development, assessing cytokine gene expression levels and TLR-4/NFκB activation mimicking neonatal NEC.

## Material and methods

### Primary antibodies

Rabbit monoclonal anti-phospho-NFκB p65 (Ser536; 93H1; #3033) and rabbit monoclonal NFκB p65 (D14E12; #8242) were purchased from Cell Signaling Technology, Danvers, Massachusetts, US. Rabbit polyclonal anti-β-actin (ab8227) antibody was obtained from Abcam, Cambridge, UK and secondary antibody from Jackson Immunoresearch (111035144, West Grove, Pennsylvania, US).

### Animals

C57BL/6 mice were obtained from the animal care facility of the Medical Faculty of Leipzig (MEZ), housed under specific pathogen-free conditions, and treated in accordance with the local animal protection legislation. All animal experiments were approved by the local ethics committee for animal experimentation of the University of Leipzig (T02/20).

### Primary cell isolation and LPS stimulation

Mice at different stages (P7, P14, P28, P56) of gastrointestinal development were sacrificed to harvest the small intestines. The intestine was flushed with ice-cold PBS, opened longitudinally and cut into 1–2 mm long pieces. These pieces were shaken for 10 min at 37 °C in HBSS (Grand Island, New York) containing 10% FCS, 1% penicillin/streptomycin (Gibco) and 100 mM DTT (Thermo Fisher, Waltham, Massachusetts, US).

For isolation of IECs, cell suspensions were sequentially passed through a 100 µm and a 30 µm pore size strainers (Miltenyi Biotec, Bergisch Gladbach, Germany) and then centrifuged for 10 min at 270 g. The pellet containing IECs was resuspended in DMEM (Gibco) supplemented with 10% FCS, 1 mM sodium-pyruvate (Gibco), 2 mM L-glutamine (Gibco) and 1% penicillin/streptomycin.

For isolation of IELs, the supernatant from the IEC fraction was incubated for 20 min in HBSS containing 10% FCS, 1% penicillin/streptomycin, and 500 mM EDTA (Gibco). The solution was passed through a 100 µm strainer and centrifuged at 450 g for 10 min. Cells were resuspended in RPMI supplemented with 10% FCS and 1% penicillin/streptomycin. Percoll (Ge Healthcare, Chicago, Illinois, US) stock solution was prepared by addition of 10% 10X PBS and 1 mM HEPES. The cell fraction was mixed with Percoll stock solution to get a final concentration of 44% Percoll. A density gradient was generated by underlying 70% Percoll. Centrifugation was carried out at 700 g for 30 min without deceleration. IELs were harvested, washed with PBS, centrifuged at 400 g for 10 min and resuspended in RPMI (Gibco) supplemented with 10% FCS, 1 mM sodium-pyruvate, 2mM L-glutamine, and 1% penicillin/streptomycin. IECs were seeded in 24-well plates at a concentration of 5 × 10⁶ cells/mL and subsequently stimulated with 1 μg/mL LPS for 3 hours. Similarly, IELs were seeded at a concentration of 2 × 10⁵ cells/mL and treated under the same conditions as the IECs.

### Real time quantitative RT-PCR

For gene expression analysis, RNA was isolated using the RNeasy plus Micro Kit (Qiagen, Venlo, The Netherlands) according to the manual’s instructions. The reverse transcriptase reaction was carried out with M-MLV RT (Invitrogen, Waltham, Massachusetts, U.S.). RT-qPCR was performed using the QuantiTect SYBR® Green RT-PCR Kit (Qiagen) on a Mastercycler Realplex 2 (Eppendorf, Hamburg, Germany). Oligonucleotides were obtained from Biomers (Ulm, Germany) and sequences are listed in Table 1. The relative expression of each gene was calculated using delta Ct values and normalised to Gapdh as the housekeeping gene.

**Table 1:** Oligonucleotide sequences used for RT-PCR.

Gene	Forward	Reverse
*Gapdh*	*TGAAGCAGGCATCTGAGGG*	*CGAAGGTGGAAGAGTGGGAG*
*Reg3γ*	*AGCTTCCTTCCTGTCCTCCA*	*CTCCCATCCACCTCTGTTGG*
*Lyz1*	*AATGGATGGCTACCGTGGTG*	*CGGTCTCCACGGTTGTAGTT*
*Si*	*ACTGGAATGCTGGAGTTCGG*	*GCTCCCAGTTGCATCCATCT*
*Tnf-α*	*TTCCGAATTCACTGGAGCCTCGAA*	*TGCACCTCAGGGAAGAATCTGGAA*
*Il-1ß*	*AGTGTGGATCCCAAGCAATACCCA*	*TGTCCTGACCACTGTTGTTTCCCA*
*Kc*	*ACCCAAACCGAAGTCATAGCC*	*TTGTCAGAAGCCAGCGTTCA*
*Il-6*	*CCAATTTCCAATGCTCTCCT*	*ACCACAGTGAGGAATGTCCA*
*Tlr-4*	*GCTTACACCACCTCTCAAAC*	*CAGCCACCAGATTCTCTAAAC*
*Md-2*	*GTTCTGCAACTCCTCCGATG*	*TCCATTGGTTCCCCTCAGTC*

### Protein purification and Immunoblotting

Cells were harvested and lysed in RIPA buffer containing 1 mM PMSF, phosphatase-inhibitor (Serva), and protease-inhibitor (Serva, Heidelberg, Germany). Protein concentration was determined using the BCA Protein Assay Kit (Pierce, Appleton, Wisconsin, US). Equal protein amounts were separated on a 10% acrylamide gel and blotted on PDVF membranes. Unspecific binding was blocked with 5% milk in TBS-T for one hour at room temperature. Primary antibodies were diluted 1:1000 and incubation was performed overnight at 4 °C. Membranes were washed and incubated with a HRP-labeled secondary antibody (1:10000; Jackson Immunoresearch) for one hour at room temperature. Detection was performed using the WesternBright Sirius ECL Kit (Advansta, San Jose, California, US) on a ChemStudio Touch 815 Imager (Analytik Jena, Germany). Densiometric quantification of Western Blot data was performed with Image J software. Band intensities corresponding to the phosphorylated form were normalised to those of the total protein and subsequently to a loading control from the same lane to account for loading variations.

### Flow cytometry and viability

1 × 10^6^ cells of the IEL fraction were stained with CD3, CD8 and CD103 (Miltenyi, Bergisch Gladbach, Germany) after FcR Block (Miltenyi, Bergisch Gladbach, Germany) according to manufacturer´s instruction. Flow Cytometric analysis was performed with LSRFortessa (BD Bioscience, Heidelberg, Germany). Live/Dead Cell discrimination was performed with propidiumiodid (Thermo Fisher, Waltham, Massachusetts, US). Data was processed using FlowJo Software.

### Linear regression

To directly compare the kinetics of IECs and IELs, we visualized the fold change as relative percentage and defined the maximum fold change of a specific gene for a cell population as hundred percent. We performed a linear regression and plotted the curves from IECs and IELs in the same graph for each cytokine.

### Statistical analysis

All data were analysed using GraphPad Prism Version 10. ANOVA analysis, Kruskal-Wallis test, Mann-Whitney *U*-Test, and Student’s *t*-Test were used where appropriate and indicated in the figure legends. Statistical significance was defined as: *p < 0.05; **p < 0.01; ***p < 0.001; All graphs are pictured with mean ± SD from at least 3 independent experiments.

## Results

**Fig. 2:**
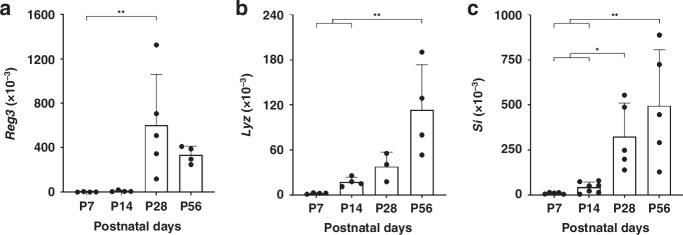
Epithelial cell-specific genes in primary IECs at different stages of gastrointestinal development. Primary murine IECs were isolated at P7 (*n* = 4–5), P14 (*n* = 4–7), P28 (*n* = 3–5), and P56 (*n* = 4–5) and gene expression of *Reg3γ* (**a**), *Lyz1* (**b**), and *Si* (**c**) was determined by RT-PCR and gene expression data was normalised to *Gapdh*. One-way ANOVA and Turkey’s multiple comparison test were used for statistical analysis (*p < 0.05, **p < 0.01).

We analysed the inflammatory response of primary isolated IECs from different gastrointestinal stages P7, P14, P28, and P56 with and without proinflammatory LPS stimulation to induce signaling via TLR-4. At first the basal expression of epithelial cell-specific genes *Regenerating family member 3 gamma* (*Reg3γ)*, *Lysozyme 1* (*Lyz1)*, and S*ucrase isomaltase* (*Si*) in primary IECs was examined (Fig. 2). *Reg3γ* is mainly expressed by Paneth cells and enterocytes. Paneth cells also display high levels of *Lyz1* expression. In both cases the expression pattern of these two genes follows the differentiation of Paneth cell during development (Fig. 2a, b). In contrast, *Si* expression is restricted to enterocytes and increases gradually throughout the gestational stages (Fig. 2c).

### Induced LPS susceptibility of IECs at early stages of gastrointestinal development

Second, we determined the expression of *Tnf-α*, *Il-1β*, *Kc* and, *Il-6* known to be induced via the LPS TLR-4 axis to address the degree of inflammatory gene response.^12–14^

**Fig. 3:**
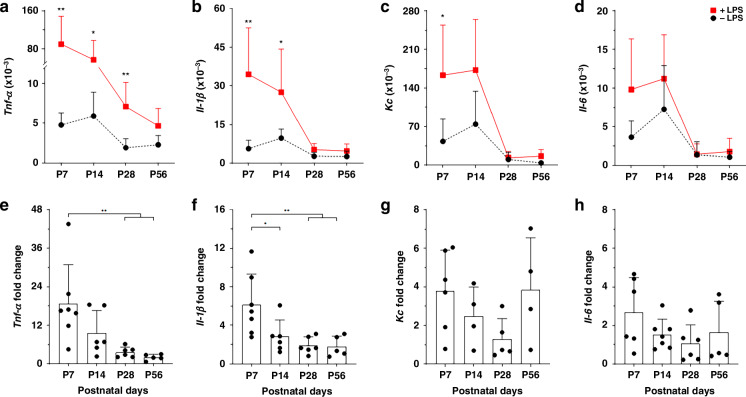
Increased LPS susceptibility of IECs at early stages of gastrointestinal development. Primary murine IECs wer*e* isolated at P7 (*n* = 6–7), P14 (*n* = 4–7), P28 (*n* = 5–6), and P56 (*n* = 4–5) and stimulated for 3 hours with 1 µg/mL LPS. RNA was isolated and gene expression of stimulated (red line; + LPS) and unstimulated (black line; - LPS) IECs from different ages for *Tnf-α* (**a**)*, Il-1β* (**b**)*, Kc* (**c**), and *Il-6* (**d**) was assessed by RT-PCR and analysed. Gene expression data was normalised to *Gapdh* as reference gene. Unstimulated and stimulated gene expression of *Tnf-α* (**a**), *Il-1β* (**b**), *Kc* (**c**), and *Il-6* (**d**) at an equal gestational stage were statistically compared by *t*-test. Fold changes of *Tnf-α* (**e**), *Il-1β* (**f**), *Kc* (**g**), and *Il-6* (**h**) expression of stimulated and unstimulated IECs over different ages were analysed by ANOVA, Turkey’s post hoc test (*p < 0.05, **p < 0.01).

We observed a significant LPS-induced increase in *Tnf-α* expression in IECs from P7 to P28, with levels stabilizing at P56 (Fig. 3a). The *Tnf-α* response decreased progressively with higher maturation, with P7 showing the highest sensitivity to LPS, while older groups exhibited reduced responses (Fig. 3e). Similar to *Tnf-α*, we observed strong LPS-induced expression of *Il-1β* and *Kc* at early developmental stages. IECs from P7 and P14 showed significant responses, while later stages were unresponsive (Fig. 3b). *Il-1β* induction was highest at P7 and P14 (Fig. 3f), and *Kc* showed a decline in induction mirroring that of *Tnf-α* and *Il-1β* with a significant response only at P7 (Fig. 3c, g). *Il-6* induction was highest at P7 (*p* = 0.052) and showed no significant induction across developmental stages (Fig. 3d, h). Thus, IECs from early stages (P7, P14) showed both, highest basal gene expression and strongest LPS-induced expression for all four genes. Notably, the level of basal gene expression did not impact on the magnitude of gene induction by LPS.

**Fig. 4:**
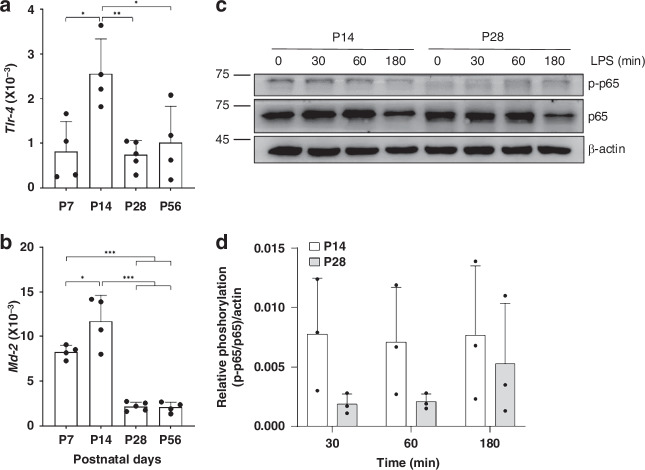
Induction of LPS-mediating signaling components at early stages of gastrointestinal development. Basal *Tlr-4* (**a**) and *Md-2* (**b**) gene expression was analysed and normalised to *Gapdh* at P7 (*n* = 4), P14 (*n* = 4), P28 (*n* = 5) and P56 (*n* = 4–5) (ANOVA, Turkey’s post hoc test; *p < 0.05, **p < 0.01, ***p < 0.001). Representative western blot analysis of phosphorylated p65 in primary murine IECs at P14 and P28 stimulated with 1 µg/mL LPS at 0, 30, 60 and 180 min after treatment (**c**). Relative densiometric quantification of phosphorylated p65 in IECs from P14 and P28 mice at different times after LPS stimulation (*n* = 3) (**d**).

Third, we studied the expression of the upstream *Tlr-4* receptor, which mediates LPS signaling. We found the highest *Tlr-4* expression in unstimulated IECs at P14 (Fig. 4a). The expression of the *Tlr-4* co-receptor *Md-2* was also elevated at P7 and P14 compared to P28 and P56 (Fig. 4b). The *Tlr-4* expression pattern matched the basal expression of proinflammatory genes, with both peaking at P14.

TLR-4 stimulation by LPS induces the phosphorylation of the NFκB subunit p65, driving inflammatory gene transcription as the primary mediator of inflammatory signaling from the cell surface to the nucleus.^7,8^ Based on the high *Tlr-4* levels at early developmental stages, we analysed p65 activation in IECs from P14 and P28 (Fig. 4c). In P14 IECs, rapid NFκB activation occurred, with phosphorylated p65 levels increasing within 30 minutes and remaining stable at 60 minutes before returning to baseline after 3 hours. In contrast, IECs from P28 showed much lower p65 phosphorylation after LPS stimulation compared to P14. While P14 IECs had basal p65 activity even without stimulation, this was absent in P28. Densiometric quantification indicated an overall higher phosphorylation level after LPS stimulation in P14 IECs compared to P28 IECs (Fig. 4d). We did not observe any effect on IEC viability after LPS exposure between P14 and P28.

Taken together, IECs at early developmental stages showed high susceptibility to LPS, with elevated proinflammatory gene expression, higher levels of *Tlr-4* and its co-receptor *Md-2*, and stronger p65 activation.

### Increased LPS-mediated induction of IELs at mature stages of gastrointestinal development

**Fig. 5:**
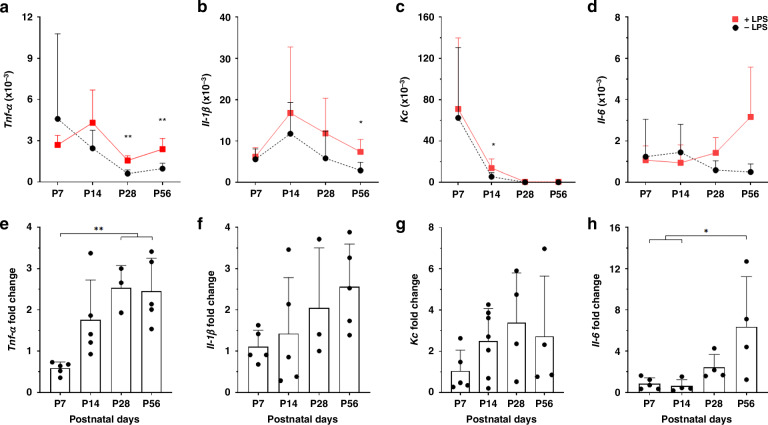
Recumbent LPS susceptibility of IELs at early stages of gastrointestinal development. Primary murine IELs were isolated at P7 (*n* = 5), P14 (*n* = 4–7), P28 (*n* = 3–4), and P56 (*n* = 4–5) and stimulated for 3 hours with 1 µg/mL LPS. RNA was isolated and gene expression of stimulated (red line; +LPS) and unstimulated (black line; -LPS) IELs from different ages for *Tnf-α* (**a**), *Il-1β* (**b**), *Kc* (**c**), and *Il-6* (**d**) was assessed by RT-PCR and analysed. Gene expression data was normalised to *Gapdh* as reference gene. Unstimulated and stimulated gene expression of *Tnf-α* (**a**), *Il-1β* (**b**), *Kc* (**c**), and *Il-6* (**d**) at an equal gestational stage were statistically compared by *t*-test. Fold changes of *Tnf-α* (**e**), *Il-1β* (**f**), *Kc* (**g**), and *Il-6* (**h**) expression of stimulated and unstimulated IELs over different ages were analysed by ANOVA, Turkey’s post hoc test (*p < 0.05, **p < 0.01).

Comparable to the protocol for IECs, gene expression of key inflammatory markers *Tnf-α*, *Il-1β*, *Kc*, and *Il-6* was analysed in both LPS-stimulated and unstimulated IELs. The basal expression of all inflammatory genes decreased from early (P7 and P14) to older stages of intestinal development (Fig. 5). However, LPS-induced *Tnf-α* expression was detected only in IELs at later developmental stages (P28 and P56), with a greater fold increase compared to P7 (Fig. 5a, e). *Il-1β* showed significant expression only at P56 (Fig. 5b, f), while *Kc* responded to LPS only at P14 (Fig. 5c, g). *Il-6* upregulation was seen at P28 and P56, though not statistically significant, with higher responsiveness at P56 (Fig. 5d, h).

**Fig. 6:**
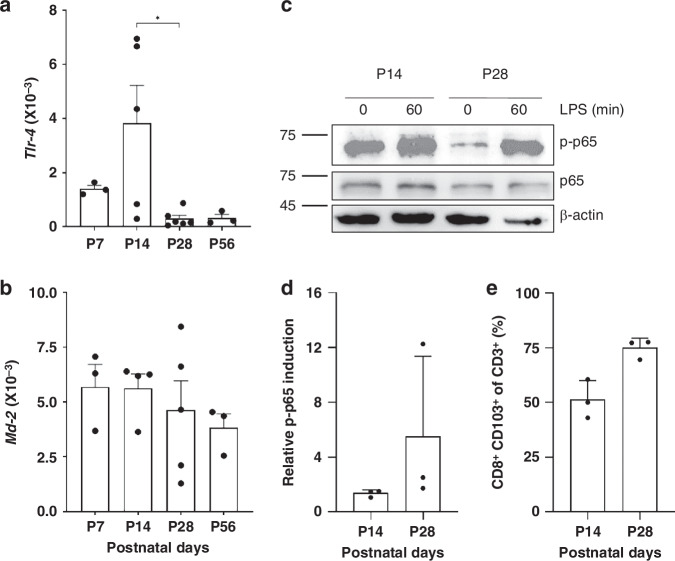
Different regulation of LPS signalling components in IELs between distinct gestational stages. Basal *Tlr-4* (**a**) and *Md-2* (**b**) gene expression was analysed and normalised to *Gapdh* at P7 (*n* = 3), P14 (*n* = 4–5), P28 (*n* = 5–6) and P56 (*n* = 3) (ANOVA, Turkey’s post hoc test; *n* = 4–6; *p < 0.05). Representative western blot analysis of phosphorylated p65 in primary murine IELs at P14 and P28 stimulated with 1 µg/mL LPS at 0 and 60 minutes after treatment (**c**). Relative densiometric quantification of phosphorylated p65 induction in IELs from P14 and P28 mice (*n* = 3) (**d**). Flow cytometric analysis of CD8 + CD103+ of CD3+ IELs at P14 and P28 (*n* = 3) (**e**).

We also analysed the mediators of LPS treatment. In unstimulated IELs, basal *Tlr-4* expression was elevated at early developmental stages (Fig. 6a) whilst the expression of its co-receptor *Md-2* remained consistent across all stages (Fig. 6b). Next, we evaluated p65 activation in IELs from P14 and P28 after LPS treatment. While P14 IELs showed higher basal p65 activity, LPS significantly increased phosphorylated p65 levels in both groups, with a greater increase in P28 IELs (Fig. 6c). Upon LPS stimulation, we observed an elevated increase in activation of p65 in P28 compared to P14 (Fig. 6d). A concurrent increase in CD3⁺CD8⁺CD103⁺ IELs was observed during the same period (Fig. 6e).

In summary, IELs at early developmental stages showed high basal proinflammatory gene expression, yet irresponsive to LPS treatment. These early stages were marked by elevated *Tlr-4* expression and high basal p65 activity. As development progressed, basal proinflammatory gene expression decreased, and IELs became responsive to LPS. This increased LPS responsiveness in later stages was accompanied by reduced basal *Tlr-4* expression and lower p65 activity, but more pronounced p65 activation after LPS stimulation.

### Shift of cytokine response to LPS stimulation in the developmental intestinal epithelial layer

**Fig. 7:**
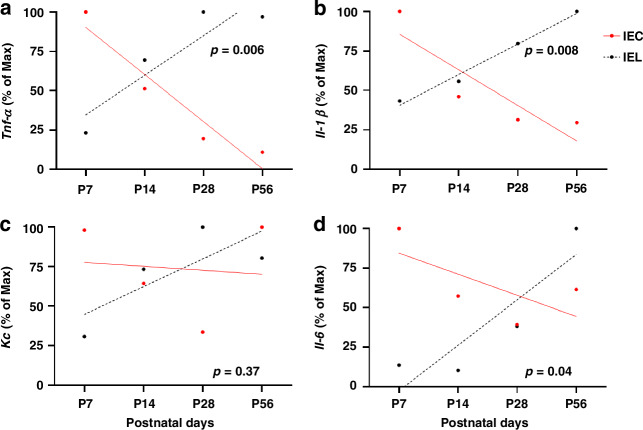
Switch of cytokine response in intestinal epithelial layer. For each specific gene, the maximum fold change was set to hundred percent, and the relative percentage over the time of gastrointestinal maturation was determined. Linear regression analysis was performed to determine statistical difference.

To compare age-dependent differences in the LPS-response of IECs and IELs for each cytokine, we performed a linear regression analysis of the gene expression levels normalised to the maximum fold change. With gastrointestinal development the expression of *Tnf-α*, *Il-1β*, and *Il-6* decreased in IECs and increased in IEL, depicting a cytokine attenuation in IECs and an induction in IELs with a shift between P14 and P28 (Fig. 7).

## Discussion

We analysed both the baseline as well as LPS-mediated proinflammatory response of murine IECs and IELs at various stages of gastrointestinal development (P7, 14, 28, and 56). This analysis was based on a detailed characterization of intestinal tissue under homeostatic conditions, with age-equivalency between mice and men as defined by Stanford and colleagues.^11^

We observed elevated basal proinflammatory cytokine expression in IECs at P7 and P14. This correlated with the *Tlr-4* expression in these groups. Expression of *Tlrs* during postnatal development has been shown to be highly dynamic and increased at these stages of development compared to older ones. Additionally, it is associated with an increased level of cytokine expression referred to as “physiological” inflammation and suggested to be a consequence of microbial colonization.^15^

IECs were highly susceptible to LPS stimulation at early and IELs at late stages of gastrointestinal maturation, accompanied by different activation level of the NFκB subunit p65. We detected a high degree of endotoxin susceptibility to LPS stimulation in IECs at early gastrointestinal stages, which declined continuously with ongoing maturation. This is consistent with others reporting that the immature state has a higher susceptibility to inflammatory insults compared to mature states.^10,16–20^ While most of these studies only compare two distinct intestinal maturation stages, our data provides a more detailed timeline. Our findings indicate a continuous decline in LPS induced proinflammatory gene expression with a higher maturation state of IECs rather than a sharp demarcation.

Previous research has shown that murine IECs acquire endotoxin tolerance shortly after birth. For example, vaginal delivery triggers a TLR-4 mediated immune response via p65 activation, making IECs less susceptible to LPS stimulation in the postnatal period in mice.^21^ This acquisition of endotoxin tolerance differs from the gradual maturation process as seen in our study. However, this does not imply that immature IECs are entirely unresponsive to postnatal inflammatory insults. Lotz and colleagues observed no proinflammatory response in 1, 6, and 28-day-old mice exposed to LPS concentrations of 1–10 ng/mL, suggesting that IECs may develop tolerance to these levels. In healthy intestines, LPS concentrations typically range from 0–1 ng/mL under non-inflammatory conditions.^22^ In contrast, our study employed an LPS concentration one hundred times higher, indicating that immature IECs may be vulnerable or may exceed their tolerance to endotoxins at significantly elevated levels. Accordingly, intestinal LPS plasma levels in experimental murine NEC models can reach up to 13 µg/mL.^22^

Our results suggest that p65 activation in IECs at P28 is not associated with an inflammatory response comparable to that observed at P14. The discrepant regulation of p65 activation may reflect inherent developmental changes in IECs that occur with maturation. Our data show that in earlier stages IECs express higher levels of basal *Tlr-4/Md-2, as we*ll as increased proinflammatory mediators at baseline and after stimulation, demonstrating heightened sensitivity to LPS. At P28, the downregulation of *Tlr-4/Md-2* and p65 activity indicates a more stable interaction with the microbiome and a shift towards an anti-inflammatory state.

Based on developmental patterns, intestinal maturation in P28 mice closely resembles that of full-term human intestines, while P14 mice correspond to preterm human at approximately 22–24 weeks of gestation.^11^ Although we did not test human specimens, the heightened baseline activity and increased susceptibility observed in immature IECs, particularly in a dysregulated microbiome modeled by LPS stimulation, parallels the higher incidence of NEC in preterm infants (9–10%) compared to those born at term.^2^

In contrast to IECs, IELs became increasingly sensitive to LPS with age, accompanied by a decrease in baseline cytokine expression. In our experiments, IELs displayed higher baseline inflammatory activity at P14, characterised by elevated basal *Tlr-4* expression and increased basal p65 activation. By P28, however, this activity diminished, corresponding with reduced *Tlr-4* expression. This reduction may enable mature IELs to respond more effectively to LPS stimulation. Prior to maturation, IELs exist in a hyperactive state where further stimulation does not amplify their activity, indicating that maturation implicates a reduction in baseline activity.

In general, IELs are predominantly CD4+ or CD8 + T-cells that can be further categorized into TCRαβ+ and TCRγδ+ cells based on their T cell receptor type. TCRαβ + IELs are classic peripheral T-cells typically involved in the adaptive immune response and recognizing antigens presented by MHC class molecules.^23^ It has been shown that IELs are reactive to LPS in vivo.^24^ Consistent with our findings, CD8αβ + TCRαβ + IELs show an age-dependent rise in IFN-γ and Granzyme B secretion upon PMA and Ionomycin activation.^25^ These IELs, almost absent at birth, increase significantly with maturation in complete small intestinal tissue, especially post-weaning.^26,27^

TCRγδ + IELs are equally important, playing a key role in preserving epithelial integrity and providing rapid immune responses to tissue stress and infection. These cells function independently of MHC antigen presentation, instead responding to tissue damage and regulating inflammation.^23^ The number of TCRγδ + IELs is very low immediately after birth but begins to increase at day 10, reaching about 50% of total IELs by day 25 in mice.^26^ Importantly, it has been shown in mouse and human that a reduction or loss of TCRγδ+ is associated with the onset of NEC.^25,28^ As TCRγδ + IELs increase in number, they may help to modulate the inflammatory environment, represented by increased baseline activity before P28 in our study, and protect against conditions like NEC.

The functional regulation of IELs during the postnatal period is also critical. In murine intestinal tissue, most CD4+ lymphocytes are intrinsically responsive but actively suppressed in their maturation during the postnatal period and capacity for antigen presentation. This inhibition is mediated directly by neonatal regulatory cells like Foxp3+ Tregs, and indirectly by breast milk-derived maternal sIgA, impairing the microbiotic migration into the intestinal epithelium.^29^ Generally, the administration of human breast milk oligosaccharides prevents experimental NEC in vivo, while breast milk itself has protective effects against NEC in preterm infants.^30–32^ Thus, the increase of certain subpopulations like TCRαβ+ and TCRγδ+ cells during later stages of gastrointestinal maturation and impaired functional properties of IELs at early developmental stages may contribute to the increased basal activity and response to LPS stimulation at mature stages as noted in our study.

The observed switch in inflammatory behavior from IECs and IELs could be attributed to the following factors: after premature delivery, IECs acquire a transient not predetermined immunological function, partly due to the developmental elevated expression of *Tlr-4* during immature stages.^9,15^ Additionally, the production of antimicrobial factors such as Reg3γ, Lyz1, and Si may mitigate heightened inflammatory responses as maturation progresses. Correspondingly, the functional activity of IELs is actively suppressed to facilitate maturation, promoting self-tolerance and reduce autoimmune reactions. This suppression is crucial in preventing excessive inflammatory responses to neonatal intestinal antigens, such as transient breast milk antigens and early commensal bacteria.^29^

The immaturity of the gastrointestinal system plays a central role in the development of NEC. In preterm infants, the underdevelopment of the intestinal tract makes them particularly susceptible to this disease. Intestinal immaturity is characterised by structural and functional underdevelopment, an immature immune response, increased intestinal permeability, and delayed development of protective cells. A key concept for therapeutic strategies might be to accelerate maturation of intestinal tissue in preterm infants.

While corticosteroids like betamethasone are administered to pregnant woman at risk of preterm birth mainly to accelerate fetal lung maturation, there is also evidence suggesting that these steroids can accelerate intestinal maturation, thereby reducing the frequency of necrotizing enterocolitis in humans.^33,34^ Experiments indicate that antenatal corticosteroid therapy helps accelerate the maturation of the intestinal barrier by reducing intestinal permeability.^35,36^ For instance, Lu and colleagues demonstrated that antennal dexamethasone treatment led to an upregulation of surfactant-D, preserving barrier integrity and reducing inflammatory conditions in a rat model of NEC.^37^

Another therapeutic strategy to promote intestinal maturation is the supplementation of infant formula with growth factors such as epidermal growth factor (EGF). EGF has a broad range of effects, and potential mechanisms in the prevention of NEC include its role in reducing inflammation and enhancing barrier function, and regulation of apoptosis.^38^

Regarding IEL maturation, several therapeutic strategies currently used in the care of preterm infants have been shown to promote IEL development. Breast milk, for example, contains high levels of transforming growth factor-β (TGF-β) which promotes the differentiation and regulatory phenotype of IELs, supporting the gut´s immune tolerance.^39^ Additionally, breast milk derived human milk oligosaccharides (HMOs) shape the gut microbiome towards beneficial species, which in turn, support the homeostatic development of IELs.^40^

These examples illustrate that current used therapeutic options and strategies are effective in accelerating the maturation of both intestinal and immune populations shifting their phenotype toward a more homeostatic and balanced interplay.^41,42^

Our study has several limitations. First, we did not directly correlate our inflammatory data obtained from murine tissue to corresponding human stages. Access to human tissue at comparable developmental stages is severely restricted due to ethical considerations and the rarity of suitable cases, particularly for healthy reference tissue. Second, we only characterised the response of single-cell cultures, thus possible interactions between IECs and IELs or epithelial and subepithelial components remains to be targeted in future studies. Furthermore, our study only analysed postnatal murine cells after vaginal delivery. The vaginal delivery itself has an effect on the responsiveness of the intestinal epithelium, which could be a bias.^21,43,44^ Finally, IELs are a very heterogeneous cell population, consisting of many different subgroups. We focused only on the general age-dependent response of IELs after stimulation but did not measure compositional changes or differences in the response of certain subpopulations. Even though many studies already described an age-dependent change of IEL subtype composition, we did not prove the causality in our results.^26–28^

In summary, we studied the maturation of intestinal epithelial cells and their response to inflammation following LPS stimulation in detail. We found that immature IECs had elevated inflammatory cytokine expression at baseline and were highly prone to inflammation mediated by an increased activation of the TLR-4/NFĸB pathway, while IELs showed higher cytokine expression and NFĸB activation in older age groups. Our model offers a simple and effective approach to study the interactions between microbiota and epithelial cells over time, shedding light on key intracellular processes in early intestinal inflammation. The shift in responsiveness from IECs to IELs correlates with reduced NEC susceptibility as the gastrointestinal system matures.

## Data Availability

The datasets generated during and/or analysed during the current study are available from the corresponding author on reasonable request.

## References

[1] Baranowski, J. R. & Claud, E. C. Necrotizing enterocolitis and the preterm infant microbiome. *Adv. Exp. Med. Biol.* **1125**, 25–36 (2019). 30680646 10.1007/5584_2018_313

[2] Clark, R. H. et al. Characteristics of patients who die of necrotizing enterocolitis. *J. Perinatol.* **32**, 199–20 (2012). 21593813 10.1038/jp.2011.65 PMC3289772

[3] Gribar, S. C. et al. Reciprocal expression and signaling of TLR4 and TLR9 in the pathogenesis and treatment of necrotizing enterocolitis. *J. Immunol.* **182**, 636–646 (2009). 19109197 10.4049/jimmunol.182.1.636 PMC3761063

[4] Abreu, M. T. et al. Decreased expression of Toll-like receptor-4 and MD-2 correlates with intestinal epithelial cell protection against dysregulated proinflammatory gene expression in response to bacterial lipopolysaccharide. *J. Immunol.* **167**, 1609–1616 (2001). 11466383 10.4049/jimmunol.167.3.1609

[5] Bein, A., Zilbershtein, A., Golosovsky, M., Davidov, D. & Schwartz, B. LPS induces hyper-permeability of intestinal epithelial cells. *J. Cell. Physiol.* **232**, 381–390 (2017). 27191060 10.1002/jcp.25435

[6] Williams, J. M. et al. A mouse model of pathological small intestinal epithelial cell apoptosis and shedding induced by systemic administration of lipopolysaccharide. *Dis. Model. Mech.* **6**, 1388–1399 (2013). 24046352 10.1242/dmm.013284 PMC3820262

[7] Chow, J. C., Young, D. W., Golenbock, D. T., Christ, W. J. & Gusovsky, F. Toll-like receptor-4 mediates lipopolysaccharide-induced signal transduction. *J. Biol. Chem.* **274**, 10689–10692 (1999). 10196138 10.1074/jbc.274.16.10689

[8] Sakurai, H., Chiba, H., Miyoshi, H., Sugita, T. & Toriumi, W. IkappaB kinases phosphorylate NF-kappaB p65 subunit on serine 536 in the transactivation domain. *J. Biol. Chem.* **274**, 30353–30356 (1999). 10521409 10.1074/jbc.274.43.30353

[9] Leaphart, C. L. et al. A critical role for TLR4 in the pathogenesis of necrotizing enterocolitis by modulating intestinal injury and repair. *J. Immunol.* **179**, 4808–4820 (2007). 17878380 10.4049/jimmunol.179.7.4808

[10] Yu, Y. et al. Increased inflammatory reaction to intestinal ischemia-reperfusion in neonatal versus adult mice. *Eur. J. Pediatr. Surg.* **25**, 46–50 (2015). 25422903 10.1055/s-0034-1387945

[11] Stanford, A. H. et al. A direct comparison of mouse and human intestinal development using epithelial gene expression patterns. *Pediatr. Res.* **88**, 66–76 (2020). 31242501 10.1038/s41390-019-0472-y PMC6930976

[12] Vinderola, G., Matar, C. & Perdigon, G. Role of intestinal epithelial cells in immune effects mediated by gram-positive probiotic bacteria: involvement of toll-like receptors. *Clin. Diagn. Lab. Immunol.* **12**, 1075–1084 (2005). 16148174 10.1128/CDLI.12.9.1075-1084.2005 PMC1235795

[13] Rizzo, V. et al. Baicalin-induced autophagy preserved LPS-stimulated intestinal cells from inflammation and alterations of paracellular permeability. *Int. J. Mol. Sci*. **22** (2021). 10.3390/ijms22052315 PMC7956379 33652555

[14] Liu, Y., Fatheree, N. Y., Mangalat, N. & Rhoads, J. M. Human-derived probiotic *Lactobacillus reuteri* strains differentially reduce intestinal inflammation. *Am. J. Physiol. Gastrointest. Liver Physiol.* **299**, G1087–G1096 (2010). 20798357 10.1152/ajpgi.00124.2010 PMC2993169

[15] Inoue, R., Yajima, T. & Tsukahara, T. Expression of TLR2 and TLR4 in murine small intestine during postnatal development. *Biosci. Biotechnol. Biochem.* **81**, 350–358 (2017). 27838962 10.1080/09168451.2016.1254534

[16] Claud, E. C., Savidge, T. & Walker, W. A. Modulation of human intestinal epithelial cell IL-8 secretion by human milk factors. *Pediatr. Res.* **53**, 419–425 (2003). 12595589 10.1203/01.PDR.0000050141.73528.AD

[17] Chan, K. L., Ho, J. C. Y., Chan, K. W. & Tam, P. K. H. A study of gut immunity to enteral endotoxin in rats of different ages: a possible cause for necrotizing enterocolitis. *J. Pediatr. Surg.* **37**, 1435–1440 (2002). 12378449 10.1053/jpsu.2002.35407

[18] Liboni, K. C., Li, N., Scumpia, P. O. & Neu, J. Glutamine modulates LPS-induced IL-8 production through IkappaB/NF-kappaB in human fetal and adult intestinal epithelium. *J. Nutr.* **135**, 245–251 (2005). 15671221 10.1093/jn/135.2.245

[19] Neal, M. D. et al. A critical role for TLR4 induction of autophagy in the regulation of enterocyte migration and the pathogenesis of necrotizing enterocolitis. *J. Immunol.* **190**, 3541–3551 (2013). 23455503 10.4049/jimmunol.1202264 PMC3608826

[20] Ginzel, M. et al. Dextran sodium sulfate (DSS) induces necrotizing enterocolitis-like lesions in neonatal mice. *PLoS ONE* **12**, e0182732 (2017). 28817583 10.1371/journal.pone.0182732 PMC5560643

[21] Lotz, M. et al. Postnatal acquisition of endotoxin tolerance in intestinal epithelial cells. *J. Exp. Med.* **203**, 973–984 (2006). 16606665 10.1084/jem.20050625 PMC2118301

[22] Guo, S., Al-Sadi, R., Said, H. M. & Ma, T. Y. Lipopolysaccharide causes an increase in intestinal tight junction permeability in vitro and in vivo by inducing enterocyte membrane expression and localization of TLR-4 and CD14. *Am. J. Pathol.* **182**, 375–387 (2013). 23201091 10.1016/j.ajpath.2012.10.014 PMC3562736

[23] McDonald, B. D., Jabri, B. & Bendelac, A. Diverse developmental pathways of intestinal intraepithelial lymphocytes. *Nat. Rev. Immunol.* **18**, 514–525 (2018). 29717233 10.1038/s41577-018-0013-7 PMC6063796

[24] Nüssler, N. C. et al. Upregulation of intraepithelial lymphocyte (IEL) function in the small intestinal mucosa in sepsis. *Shock* **16**, 454–458 (2001). 11770044 10.1097/00024382-200116060-00009

[25] Mayassi, T. & Jabri, B. Human intraepithelial lymphocytes. *Mucosal Immunol.* **11**, 1281–1289 (2018). 29674648 10.1038/s41385-018-0016-5 PMC6178824

[26] Steege, J. C., Buurman, W. A. & Forget, P. P. The neonatal development of intraepithelial and lamina propria lymphocytes in the murine small intestine. *Dev. Immunol.* **5**, 121–128 (1997). 9587712 10.1155/1997/34891 PMC2275980

[27] Manzano, M., Abadía-Molina, A. C., García-Olivares, E., Gil, A. & Rueda, R. Absolute counts and distribution of lymphocyte subsets in small intestine of BALB/c mice change during weaning. *J. Nutr.* **132**, 2757–2762 (2002). 12221241 10.1093/jn/132.9.2757

[28] Weitkamp, J. H. et al. Small intestinal intraepithelial TCRγδ+ T lymphocytes are present in the premature intestine but selectively reduced in surgical necrotizing enterocolitis. *PLoS ONE* **9**, e99042 (2014). 24905458 10.1371/journal.pone.0099042 PMC4048281

[29] Torow, N. et al. Active suppression of intestinal CD4+TCRαβ+ T-lymphocyte maturation during the postnatal period. *Nat. Commun.* **6**, 7725 (2015). 26195040 10.1038/ncomms8725 PMC4518322

[30] Cristofalo, E. A. et al. Randomized trial of exclusive human milk versus preterm formula diets in extremely premature infants. *J. Pediatr.* **163**, 1592–5.e1 (2013). 23968744 10.1016/j.jpeds.2013.07.011

[31] Sodhi, C. P. et al. The human milk oligosaccharides 2′-fucosyllactose and 6′-sialyllactose protect against the development of necrotizing enterocolitis by inhibiting toll-like receptor 4 signaling. *Pediatr. Res.* **89**, 91–101 (2021). 32221473 10.1038/s41390-020-0852-3 PMC7529714

[32] Ginzel, M. et al. The viral dsRNA analogue poly(I:C) induces necrotizing enterocolitis in neonatal mice. *Pediatr. Res.* **79**, 596–602 (2016). 26679153 10.1038/pr.2015.261

[33] Bauer, C. R. et al. A decreased incidence of necrotizing enterocolitis after prenatal glucocorticoid therapy. *Pediatrics* **73**, 682–688 (1984). 6371696

[34] Halac, E. et al. Prenatal and postnatal corticosteroid therapy to prevent neonatal necrotizing enterocolitis: a controlled trial. *J. Pediatr.* **117**, 132–138 (1990). 2196355 10.1016/s0022-3476(05)72461-6

[35] Israel, E. J., Schiffrin, E. J., Carter, E. A., Frieberg, E. & Walker, W. A. Cortisone strengthens the intestinal mucosal barrier in a rodent necrotizing enterocolitis model. *Adv. Exp. Med. Biol.* **310**, 375–380 (1991). 1809013 10.1007/978-1-4615-3838-7_48

[36] Israel, E. J., Schiffrin, E. J., Carter, E. A., Freiberg, E. & Walker, W. A. Prevention of necrotizing enterocolitis in the rat with prenatal cortisone. *Gastroenterology* **99**, 1333–1338 (1990). 2210242 10.1016/0016-5085(90)91158-3

[37] Lu, L., Lu, J., Yu, Y. & Claud, E. Necrotizing enterocolitis intestinal barrier function protection by antenatal dexamethasone and surfactant-D in a rat model. *Pediatr. Res.* **90**, 768–775 (2021). 33469185 10.1038/s41390-020-01334-0 PMC8566228

[38] Coursodon, C. F. & Dvorak, B. Epidermal growth factor and necrotizing enterocolitis. *Curr. Opin. Pediatr.* **24**, 160–164 (2012). 22227788 10.1097/MOP.0b013e3283504ddb

[39] Konkel, J. E. et al. Control of the development of CD8αα+ intestinal intraepithelial lymphocytes by TGF-β. *Nat Immunol.* **12** 312–9 (2011). 10.1038/ni.1997 PMC3062738 21297643

[40] Charbonneau, M. R. et al. Sialylated milk oligosaccharides promote microbiota-dependent growth in models of infant undernutrition. *Cell* **164**, 859–871 (2016). 26898329 10.1016/j.cell.2016.01.024 PMC4793393

[41] Patel, R. M. & Denning, P. W. Intestinal microbiota and its relationship with necrotizing enterocolitis. *Pediatr. Res.* **78**, 232–238 (2015). 25992911 10.1038/pr.2015.97 PMC4655440

[42] Fu, Y. et al. Risk factors for necrotizing enterocolitis associated mortality. *Pediatr. Med.* **3**, 2 (2020).

[43] Guthrie, S. O. et al. Necrotizing enterocolitis among neonates in the United States. *J. Perinatol.* **23**, 278–285 (2003). 12774133 10.1038/sj.jp.7210892

[44] Zachariassen, L. F. et al. Cesarean section increases sensitivity to oxazolone-induced colitis in C57BL/6 mice. *Mucosal Immunol.* **12**, 1348–1357 (2019). 31554900 10.1038/s41385-019-0207-8

